# COMPARISON OF SEDENTARY BEHAVIOR GUIDELINES FOR OLDER ADULTS: A REVIEW OF LITERATURE AND QUALITY APPRAISAL

**DOI:** 10.1093/geroni/igad104.2843

**Published:** 2023-12-21

**Authors:** Amy Huang, Ellen Wang, Stephanie Sanger, Alexandra Papaioannou, Isabel Rodrigues

**Affiliations:** McMaster University, Hamilton, Ontario, Canada; University of British Columbia, Vancouver, British Columbia, Canada; McMaster University, Hamilton, Ontario, Canada; McMaster University, Hamilton, Ontario, Canada; McMaster University, Hamilton, Ontario, Canada

## Abstract

Most older adults (≥65 years) accumulate >8.5 hours/day of continuous sedentary time, which is associated with increased risk of metabolic syndromes and falls. The impact of increased sedentary time in older adults globally has prompted the development of national and international sedentary behaviour guidelines. The purpose of our review was to identify and compare national and international sedentary behaviour guidelines for older adults and appraise the quality of the guidelines to promote best practice in guideline development. We conducted our search in Medline, Embase, Global Health and relevant grey literature. We included the most updated guidelines for older adults written in English. AGREE II was used to assess the quality of the recommendations. We identified eight national and international sedentary behaviour guidelines for older adults. The guidelines were developed from reviews, cohort studies, knowledge user opinions, or other guidelines; all guidelines were based on low quality and certainty of evidence. The terms “sedentary behaviour” and “sedentary time” were used interchangeably, and the definitions of both terms were not consistent between guidelines. Six guidelines recommended a reduction in total time with one suggesting limiting sedentary time to < 8 hours/day. Three guidelines suggested reallocating sedentary time to light activity, with one recommending to stand-and-stretch every hour for 5-minutes. Most sedentary behaviour guidelines for older adults are based on low quality and low certainty evidence. Terminology, definitions, and recommendations were not consistent between guidelines. Further work is needed to develop evidenced-based recommendations specific to older adults.

